# Acceleration of Image Segmentation Algorithm for (Breast) Mammogram Images Using High-Performance Reconfigurable Dataflow Computers

**DOI:** 10.1155/2017/7909282

**Published:** 2017-05-22

**Authors:** Ivan L. Milankovic, Nikola V. Mijailovic, Nenad D. Filipovic, Aleksandar S. Peulic

**Affiliations:** ^1^Faculty of Engineering, University of Kragujevac, Sestre Janjic 6, Kragujevac, Serbia; ^2^Research and Development Center for Bioengineering, BioIRC, Prvoslava Stojanovica 6/1, Kragujevac, Serbia

## Abstract

Image segmentation is one of the most common procedures in medical imaging applications. It is also a very important task in breast cancer detection. Breast cancer detection procedure based on mammography can be divided into several stages. The first stage is the extraction of the region of interest from a breast image, followed by the identification of suspicious mass regions, their classification, and comparison with the existing image database. It is often the case that already existing image databases have large sets of data whose processing requires a lot of time, and thus the acceleration of each of the processing stages in breast cancer detection is a very important issue. In this paper, the implementation of the already existing algorithm for region-of-interest based image segmentation for mammogram images on High-Performance Reconfigurable Dataflow Computers (HPRDCs) is proposed. As a dataflow engine (DFE) of such HPRDC, Maxeler's acceleration card is used. The experiments for examining the acceleration of that algorithm on the Reconfigurable Dataflow Computers (RDCs) are performed with two types of mammogram images with different resolutions. There were, also, several DFE configurations and each of them gave a different acceleration value of algorithm execution. Those acceleration values are presented and experimental results showed good acceleration.

## 1. Introduction

Image segmentation is the most common procedure applied in medical imaging analysis. It is also one of the most important tasks in image processing [1]. In computer vision, image segmentation is the process of partitioning the image into multiple segments. This technique or group of techniques refers to dividing images into regions with similar attributes. The attributes are often gray levels, colors, edges, texture characteristics, or spectral properties. The main goal of image segmentation is to simplify the representation of an image into something that is more meaningful, thus making an image easier to analyze in the image processing tasks.

The process of image segmentation is one of the main parts in various applications in medical diagnostics. Algorithms for image segmentation are used for detecting microcalcification in mammography images [2] whose major role is in the opportune detection and treatment of a lesion. Kallergi [3] created automated computed tools for microcalcification detection based on wavelet filters and used artificial neural networks. For tumor region extraction and enhancement of the classification of mammographic images, edge detection operators are considered [4]. In order to segment dense areas of the breast and the existence of mass and to visualize other breast regions, the graph cuts (GC) segmentation technique is used [5]. Aghdam et al. [6] proposed a probabilistic approach for breast boundary extraction in mammograms.

Detection of microcalcification is, in most cases, performed with preprocessed images. Oliver et al. [7] filtered images by different kinds of filters with the aim of creating a huge dictionary database. New images were compared with a database where every pixel of the breast image is a center of the patch. The results are probability images where a higher brightness pixel corresponds to more reliance to be microcalcification.

The final result of the segmentation process is a uniform region. According to [8], the segmentation quality is rated by the ratio of uniformity and homogeneity of the estimated region. The regions need to be shell-free and edges of regions are smooth and space accurate.

The segmentation of images has a wide/significant/important application in digital mammography. The main goals of scientists and researchers are to develop sophisticated image analysis tools that can automatically detect suspicious mass regions of the breast. This process begins with extraction of regions of interest (ROIs) of breast images. Then, the detection of suspicious regions and their classification are performed, after which the comparison with the existing image database starts.

For medical diagnostic decision systems, it is very important to provide a large training data set. For preparation of this data, a large database of medical images can be very helpful. The processing time can be a limiting parameter here. To accelerate the processing time, multicore, many-core, and FPGA based dataflow architectures can be used.

In this paper, the FPGA based dataflow architecture provided by Maxeler is used to accelerate region-of-interest based image segmentation algorithm for (breast) mammogram images which is developed by Milosevic et al. [9] and described in this paper.

## 2. Background

Combining the architecture and techniques from High-Performance Computing (HPC) systems with those of RDC systems, a great increase can be achieved in calculation speed in many algorithms [10]. By familiarizing with the RDC systems and clarifying speedup possibilities, one can achieve additional performance advantages over other single-core solutions. The RDC systems represent the combination of reconfigurable logic and, in most cases, are based on FPGA architecture.

### 2.1. Field Programmable Gate Arrays

Field programmable gate arrays are integrated circuits designed in such manner so that they can be configured by the designer to implement some digital computations. They were invented in the 1980s by Santo [11]. Their main goal is to accelerate certain calculation tasks with respect to the single processing unit. They also have low processing power consumption. They achieve the best results in speedup with algorithms with limited data dependencies and significant scope for parallel execution.

In order to implement complex computational tasks, the modern FPGAs have a large number of logic blocks, I/O pads, and routing channels. The process of mapping an algorithm into FPGA requires paying special attention to the available amount of resources. The number of the logic blocks and I/O pads can be easily determined from the design, but the number of routing channels may vary significantly.

To define the behavior of the FPGA, the designer can use hardware description language (HDL) or some other specialized computer language. The design, described using some of those languages, is transformed into technology-mapped netlist via an electronic design automation tool. That netlist is then mapped to the actual FPGA architecture. At each point of the process of defining the behavior of the FPGA, the functionality of the design is validated via timing analysis, simulation, and other verification methodologies. When the process of validation is done and it is concluded that functionality of the design is correct, the binary file is generated. This binary file is then used to configure the FPGA. In theory, any algorithm can be mapped into FPGA, but, in practice, the main constraints are already mentioned: available resources, clock rates, and available I/O pads.

### 2.2. Dataflow Computing

The computers based on von Neumann architecture or control flow architecture fetch the instructions and data from the memory, perform operations specified by instructions, and write the results back to the memory. The main drawback of this architecture is that each execution of an instruction requires cyclic access for memory which results in a large number of data transfers between the Central Processing Unit (CPU) and memory.

The dataflow computing paradigm is fundamentally different from the standard control flow computing. In dataflow computing, the high level language is used to generate a dataflow graph which is directly mapped to the dataflow engine (DFE). Each node in this graph performs a specific operation and outputs the result of that operation to another node in the graph, and thus the cyclic access for memory is avoided.

Dataflow computing paradigm as such has some advantages and some disadvantages with respect to control flow computing. The main advantage is that the instructions are executed in a natural sequence as data propagate through the algorithm. It also reduces the effect of memory access latency because all of the data travel through the graph and they are attached to their nodes. The main disadvantage is the lack of central control, because each node is activated only when all of its inputs are available. Also, the nodes are forced to use data when they are available even if they are not needed at that time.

Dataflow computing is nowadays used in a large number of applications. It not only accelerates the applications, but also makes them more energy efficient compared to sequential computing because of the low processing power consumption of DFEs. Energy efficiency is achieved with low DFE's frequency and it is a well-known fact that power consumption is directly proportional to frequency. The DFE's frequency can go up to a few hundreds of MHz, while frequency of today's processors goes up to a few GHz.

This dataflow computing method is used in automation applications [12], digital signal processing [13, 14], mathematics for solving systems of equations [15], floating-point matrix multiplication [16], financial derivatives pricing [17], artificial neural networks [18], and much more.

### 2.3. High-Performance Reconfigurable Dataflow Computers

**Figure 1:**
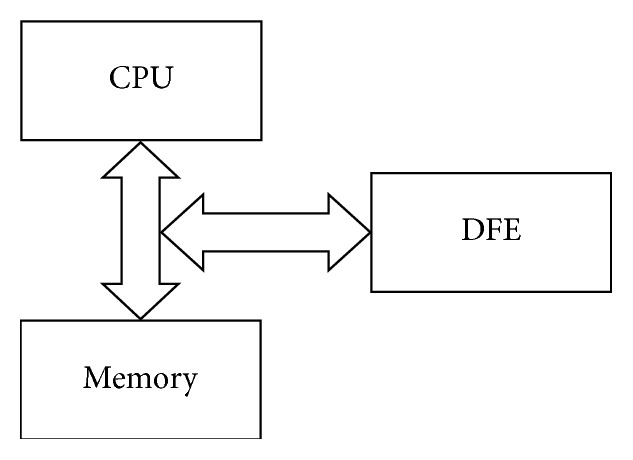
Simple HPRDC architecture.

High-Performance Reconfigurable Dataflow Computers (HPRDCs) represent the architecture that integrates the Reconfigurable Dataflow Computers and general-purpose processors or some parallel computing systems. The basic idea of HPRDCs was born in 1960 by Estrin who proposed the concept of a computer made of a general-purpose processor and reconfigurable hardware [19]. This heterogeneous architecture is mostly used in some scientific researches and in supercomputing. It is also used in industrial applications such as MORPHEUS project [20]. The simplified view of this architecture is shown in Figure 1.

One or more DFEs are interconnected via a dedicated high-speed network with the CPU and its memory. Data arrays from the CPU are transferred through this network to the DFE and streamed through DFE computational logic (i.e., DFE kernels). The results of this computation are transferred back to the CPU memory. In the HPRDC architecture, the DFE is used as an application specific processor, which accelerates some computationally expensive section of the code.

### 2.4. Maxeler's Dataflow Engines

**Figure 2:**
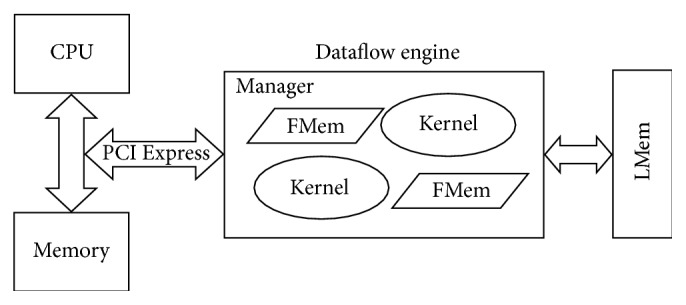
Maxeler's DFE architecture.

Maxeler has developed DFEs which use a high-speed, large area FPGA chip as a computational unit. The general architecture of these DEFs is shown in Figure 2. Each DFE consists of one or more kernels, manager, fast memory (FMem), and large memory (LMem) and is connected to the CPU via PCI Express bus.

Kernels represent hardware implementation of an algorithm and their main task is to perform computation as data flows through the DFE. The manager has a task to define the way in which data flows between kernels, memories, and host processor. The DFE has two types of memory: fast memory (FMem) and large memory (LMem). The FMem is an on-chip memory and can store several megabytes of data with terabytes/second of access bandwidth. The LMem is an off-chip memory and it is much larger than the FMem. It can store many gigabytes of data.

Maxeler has also developed its own hardware description language called MaxJ. The MaxJ is a Java based language which enables a user without significant expertise in hardware design to write high-performance applications for FPGA with a higher level of abstraction from hardware than a HDL. MaxCompiler uses MaxJ code and in its runtime generates VHDL which is built into bitstream using FPGA vendor's toolchain. Different generations of Maxeler's DFEs have different FPGA vendors which are either Xilinx or Altera. Voss et al. [21] showed on the gzip design example that using MaxJ takes only one person and a period of one month to develop an application and achieve better performance than the related work created in Verilog and OpenCL. Maxeler has also provided simulation and debugging tools which allow designs to be tested before building for a real DFE which provides much faster development than with using low level hardware description languages such as Verilog and VHDL.

Maxeler's DFEs are widely used in many fields. Gan et al. [22] summarize their experiences of using Maxeler's DFEs to eliminate the main bottlenecks and obtain higher efficiencies in solving geoscience problems. They managed to achieve better results in both performance and power efficiency over traditional multicore and many-core architectures. Grull and Kebschull [23] showed acceleration of 200 compared to an Intel i5 450 CPU for localization microscopy and acceleration of 5 over an Nvidia Tesla C1060 for electron tomography while maintaining full accuracy using Maxeler's DFEs. Gan et al. [24] used Maxeler's DFEs to find the solution of global shallow water equations (SWEs), which are one of the most essential equation sets describing atmospheric dynamics. They achieved speedup of 20 over a fully optimized design on a CPU rack with two eight-core processors and speedup of 8 over the fully optimized Kepler GPU design. They also managed to have 10 times higher power efficiency than a Tianhe-1A supercomputer node. Weston et al. [17] achieved acceleration over 270 times faster than a single Intel Core for a multiasset Monte Carlo model.

## 3. Region-of-Interest Based Image Segmentation Algorithm for (Breast) Mammogram Images

The method for mammogram ROI detection [9] is composed of pectoral muscle removal and background removal which represent any artifact present outside the breast area, such as patient markings [25]. There are two views of breast mammogram images: left sided and right sided. For the sake of simplicity, both algorithms, the background removal and the pectoral muscle removal, will be explained for the right sided view of a breast mammogram image. The algorithms for the left sided view are very similar to the algorithms for the right sided view, and thus there is no need to explain both algorithms.

### 3.1. Background Partition Removal Algorithm

The basic idea of background partition removal algorithm is to find the largest area of connected nonblack pixels and then set all other pixels to black. The algorithm is as shown in Algorithm 1.

**Figure 3:**
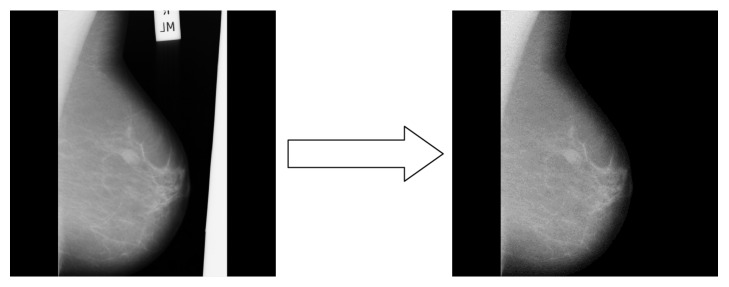
Background partition removal algorithm result.

The result of the background partition removal algorithm is shown in Figure 3. As it can be noticed, the unnecessary background has been removed successfully.

After the background partition removal has been done, the next task is pectoral muscle removal.

### 3.2. Pectoral Muscle Removal

Pectoral muscle tissue is usually denser than the rest of the breast. Therefore, pectoral muscle and the central part of the breast can be extracted by applying local threshold operation with appropriate threshold value. The algorithm for pectoral muscle removal is as shown in Algorithm 2.

**Figure 4:**
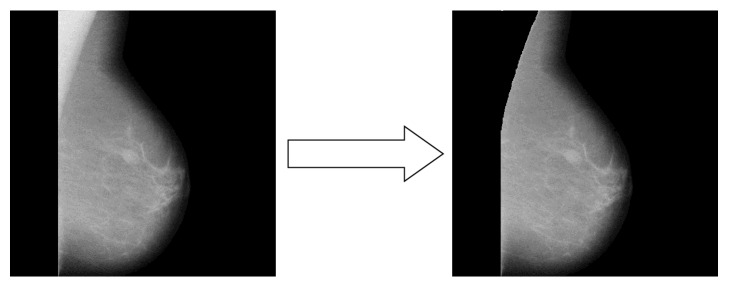
Pectoral muscle removal algorithm result.

The result of the pectoral muscle removal algorithm is shown in Figure 4. As it can be noticed, the unwanted pectoral muscle has been removed successfully.

After the pectoral muscle removal, the process of ROI extraction is performed. The breast mammogram image obtained in this way is more convenient for further processing.

## 4. Mapping the Region-of-Interest Based Image Segmentation Algorithm for (Breast) Mammogram Images on DFE

As a DFE platform for mapping the region-of-interest based image segmentation algorithm, Maxeler's DFE is used. In the case of this algorithm, the graph that represents it consists of two kernels: one for background partition removal and the other for pectoral muscle removal. The manager is responsible for getting the data about mammogram images from the host processor, streaming them to the input of the kernel for background partition removal, getting the output of this kernel and streaming it to the input of the kernel for pectoral muscle removal, and streaming the output of this kernel back to the host processor.

**Figure 5:**
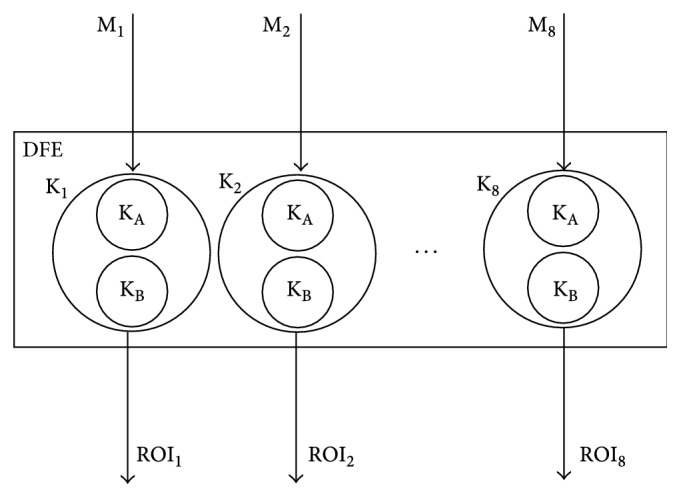
Block diagram for the DFE design.

After implementing this graph on DFE, there were still a lot of unused FPGA resources left on it. Because of that, as it is shown in Figure 5, this graph is multiplied eight times, so that the DFE can process eight mammogram images at the same time. There cannot be more than eight graphs on the DFE because it is limited to eight input and eight output streams.

The host processor streams the data about one or more (up to eight) mammogram images (M_(1–8)_) to the DFE. The manager on the DFE collects these data and streams them to the kernels K_(1–8)_. The kernels process these data and stream region-of-interest images (ROI_(1–8)_) to the manager, as a result of that processing. The manager collects these output data from the kernels and streams them to the host processor. The host processor writes these data to the memory. In this way, the process of region-of-interest based image segmentation on the DFE is done.

On the block diagram that represents the DFE design, there are eight kernels K_(1–8)_ which can process eight mammogram images at the same time. Each kernel K_(1–8)_ is constructed by two kernels: K_A_ and K_B_. The kernel K_A_ implements the background partition removal algorithm, whereas the kernel K_B_ implements the pectoral muscle removal algorithm.

**Figure 6:**
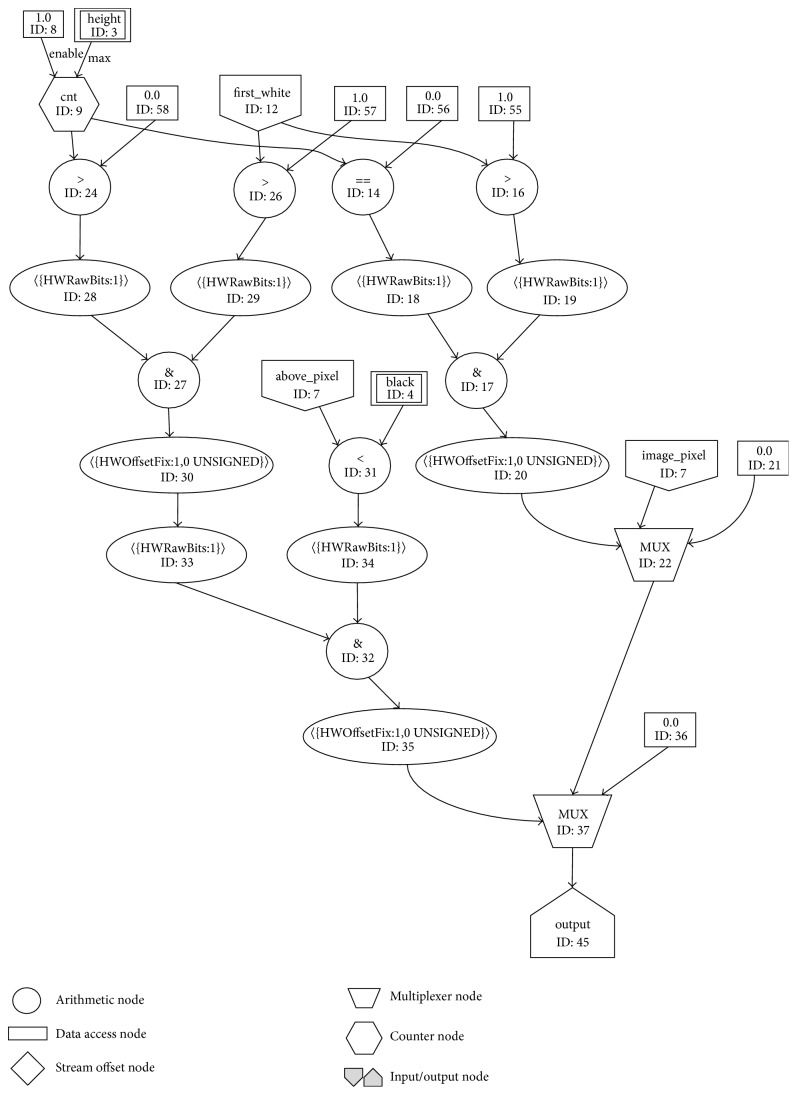
Graph for background partition removal kernel.

**Figure 7:**
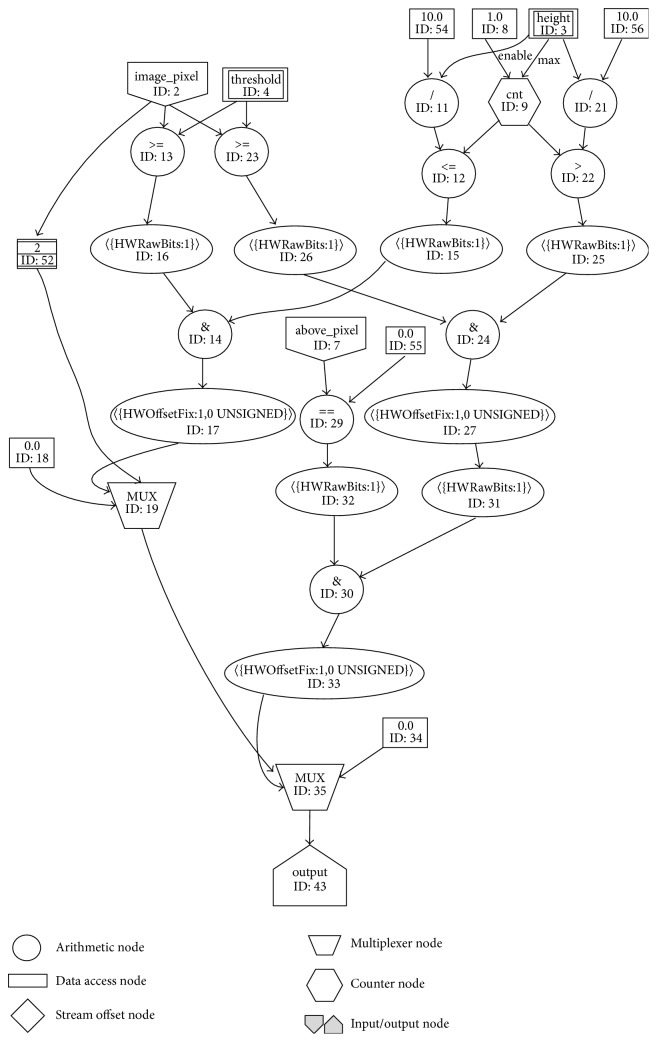
Graph for pectoral muscle removal kernel.

Both of these kernels are defined with separate graphs which describe their functionality. The simplified versions of these two graphs are presented in Figures 6 and 7. In these figures, some variables like “first_white” and “above_pixel” are presented as input streams, but in the final application they are calculated.

For the purpose of understanding the graph, they can be presented as input streams because their calculation does not have an effect on anything else and their name clearly describes what they are used for. The variable “first_white” is used to determine the first appearance connected nonblack pixels for the current row of the breast mammogram image. The variable “above_pixel” holds the value of the pixel that is in the same column as the current pixel of the breast mammogram image, but it is in the upper row. In this way, much more simplified and clearer graphs are derived.

Also, both graphs need to meet some conditions from the DFE usage point of view. They need to be designed in a way so that they use the least possible number of nodes, but also to meet the requirements of algorithms for background partition removal and pectoral muscle removal.

### 4.1. Graph for Background Partition Removal Kernel

The main task that the background partition removal kernel needs to accomplish is to remove the unnecessary background from the breast mammogram image. In Figure 6, the graph designed for the background partition removal algorithm is shown.

The graph designed for this kernel consists of two multiplexers, few arithmetic nodes, one counter node which is used to count from 0 to the number of rows of mammogram image minus 1 with Step 1, few input streams and scalar inputs, and one output stream.

The main parts of this graph are two multiplexers with IDs 22 and 37. Depending on certain conditions, they stream out the value of the current image pixel or set that value to the black and stream it to the output.

The first multiplexer (ID: 22) checks two conditions: whether the current pixel belongs to the first row of the mammogram image and whether it does not belong to the first connected part of the nonblack pixels of the first row. If these two conditions are satisfied, it sets the current pixel to the black, in which way the background partition pixel is removed, and streams it to the second multiplexer (ID: 37) and further to the output. Otherwise, if the conditions are not satisfied, the first multiplexer streams the unchanged current image pixel to the second multiplexer for processing, because it does not belong to the background partition part of the mammogram image.

The second multiplexer (ID: 37) checks whether the current pixel does not belong to the first row and to the first connected part of the nonblack pixels of the current row of the mammogram image and whether the above pixel value is smaller than the predefined value “black.” The predefined value “black” is used as a threshold for determining the color of the pixel: black or nonblack. If all the conditions are satisfied, the second multiplexer sets the current pixel to black, in which way the background partition is removed, and streams it to the output. Otherwise, if the conditions are not satisfied, the second multiplexer streams the unchanged current image pixel to the output, because it does not belong to the background partition part of the mammogram image.

The output of the kernel for the background partition removal is connected to the input of the kernel for the pectoral muscle removal.

### 4.2. Graph for Pectoral Muscle Removal Kernel

The main task that the pectoral muscle removal kernel needs to accomplish is to remove the part of the mammogram image that represents pectoral muscle tissue. In Figure 7, the graph designed for the pectoral muscle removal algorithm is shown.

The graph for background partition removal kernel consists of almost the same nodes as the graph for background partition removal. It consists of two multiplexers, several arithmetic nodes, one counter node which counts from 0 to the number of rows of mammogram image minus 1 with Step 1, few input streams and scalar inputs, and one output stream.

The main parts of this graph are two multiplexers with IDs 19 and 35. Depending on certain conditions, they stream out the value of the current image pixel or set that value to black and stream it to the output.

The first multiplexer (ID: 19) checks whether the current pixel belongs to the row that is in the first tenth part of the mammogram image and whether the current image pixel value is greater than or equal to the predefined variable “threshold.” The predefined value “threshold” is used as a threshold for determining whether the current pixel belongs to the pectoral muscle tissue part of the mammogram image. If all of these conditions are satisfied, the first multiplexer sets the current pixel to black, in which way the pectoral muscle part is removed, and streams it to the other multiplexer (ID: 35) and further to the output with no dependencies with the conditions for the other multiplexer. Otherwise, if the conditions are not satisfied, the first multiplexer streams the unchanged current image pixel to the second multiplexer for processing, because it does not belong to the pectoral muscle tissue part of the mammogram image.

The second multiplexer (ID: 35) checks whether the current pixel does not belong to the row that is in the first tenth part of the mammogram image, if the current image pixel value is greater than or equal to the predefined variable “threshold,” and if the above pixel is black. If all those conditions are satisfied, the second multiplexer sets the current pixel to black, in which way the pectoral muscle is removed, and streams it to the output. Otherwise, if the conditions are not satisfied, the second multiplexer streams the unchanged current image pixel to the output, because it does not belong to the pectoral muscle tissue part of the mammogram image.

The output of the kernel for the pectoral muscle removal is streamed to the host processor which writes it to the memory. With storing these data into the memory, the process of ROI extraction is done.

### 4.3. Resource Usage

All inputs and outputs to both above kernels have 32-bit width. Multiplexers in both kernels are 2-to-1 multiplexers with 32-bit width inputs and output and 1-bit width selection signal. The counters have also 32-bit width. Arithmetic nodes used for comparisons (<, >, ==, >=, <=) have 32-bit width inputs and 1-bit width output, AND arithmetic node has 1-bit width input and output, and deviation arithmetic node is used for 32-bit width deviation of unsigned integers.

The FPGA resource usage per each operator according to the MaxCompiler's resource annotation [26] is presented in Table 1.

## 5. Implementation Results and Discussions

As it is already mentioned, the algorithm for region-of-interest based image segmentation is mapped on Maxeler's DFE. Maxeler's DFE was chosen because, in the literature, it showed better performances and energy efficiency against desktop processors and computing servers. On the other hand, simple array of FPGAs is not chosen because languages such as Verilog and VHDL which are widely used to design FPGA require significant expertise and considerable design efforts which is opposed to Maxeler's solution which requires knowledge of Java based MaxJ only.

The DFE is attached to the host processor via PCI Express bus and it is configured with two kernels and a manager. The Maxeler dataflow computer can be understood as a combination of two programming paradigms: control flow and dataflow. Before one begins programming DFE, he/she must split the whole algorithm into its control flow and dataflow part. For instance, in this case, the control flow part consists of reading the mammogram images from the memory and writing the processed images back to the memory, whereas the dataflow part relates to the whole image segmentation algorithm.

The execution speeds of the algorithm for region-of-interest based image segmentation on a general-purpose processor and Maxeler's DFE are compared. Maxeler's DFE which was used is MAX2336B which contains Xilinx Virtex 5 XC5VLX330T FPGA chip. The comparisons were made on various configurations of DFE and with two types of mammogram images: with resolution of 1024 × 1024 pixels and 4800 × 6400 pixels.

The general-purpose processor that was used is Intel Core i3-3240 which works at a frequency of 3.40 GHz. The operating system of the machine with this processor was CentOS release 5.10. The code was written in C programming language and compiled with GCC compiler.

The DFE was, as it is already mentioned, configured in various ways. It was configured to work with only one picture at a time and with two and more, but up to eight, pictures at the same time. This was accomplished by mapping only one kernel for region-of-interest based image segmentation and then mapping two and more, but up to eight, kernels on the DFE. The DFE was also configured to work with different frequencies: 75 MHz, which is the default frequency, and 200 MHz. The code for Maxeler's DFE was written in MaxIDE development environment and was compiled using MaxCompiler [26]. The resource usage of the DFE for the case of eight kernels and frequency of 200 MHz is shown in Table 2. As it can be noticed from the table, there are still a lot of unused FPGA resources on the DFE.

**Figure 8:**
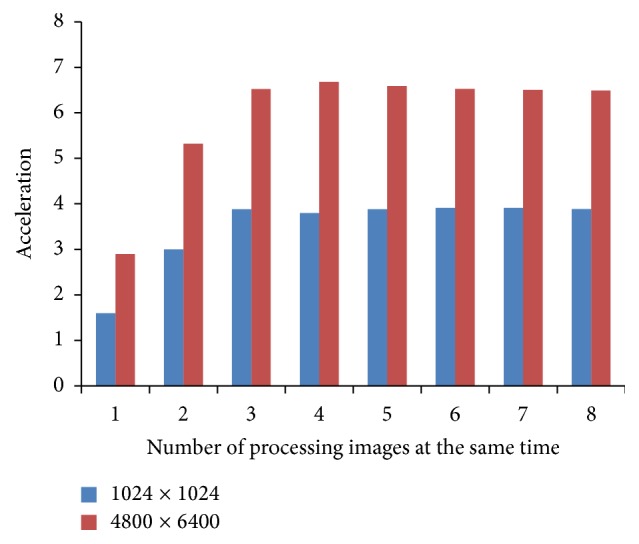
Acceleration analysis with various configurations of DFE with frequency of 75 MHz.

In Figure 8, the diagram shown presents the amount of acceleration for two types of images, with resolutions of 1024 × 1024 pixels and 4800 × 6400 pixels, and the different number of processing images at the same time.

Figure 8 displays that the acceleration is much greater if DFE processes larger mammogram images. Also, with the increase in the number of processing images at the same time, the acceleration increases to some point for both mammogram images types. From that point onwards, the acceleration is approximately constant. This is the point where acceleration gets bound with PCI Express bandwidth.

**Figure 9:**
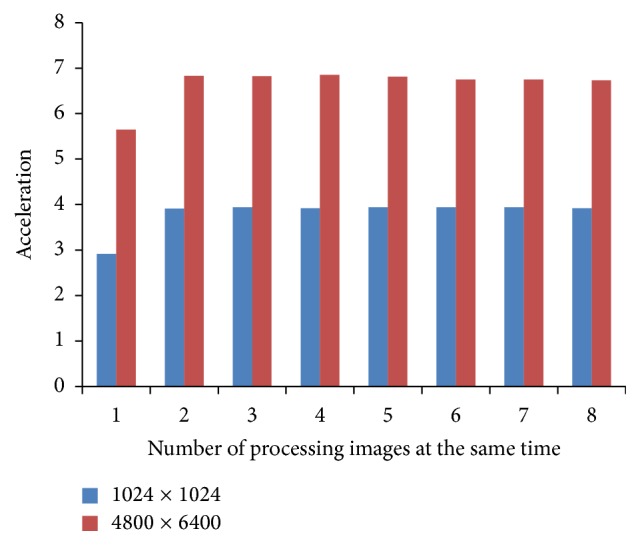
Acceleration analysis with various configurations of DFE with frequency of 200 MHz.

The same diagram as in Figure 8 is presented in Figure 9, but the frequency is greater and it is set to 200 MHz. As it can be noticed, the acceleration is still much greater if DFE processes larger mammogram images.

Compared to the acceleration results in Figure 8 with a frequency of 75 MHz, the acceleration results in Figure 9 with frequency of 200 MHz in the area of one and two processing mammogram images at the same time are much better, but outside that area the results are pretty much the same. The reason for this is also that this is the point where acceleration gets bound with PCI Express bandwidth.

The point where acceleration gets bound with PCI Express bandwidth is pretty easy to calculate. It is the point where the time required to stream data to/from the DFE starts to be greater than the time required to execute the real processing of the data. The time to process all the data can be calculated using the following formula:

(1) $$\begin{array}{c} T_{\text{processing}}=\frac{\text{cycles}}{\text{frequency}}. \end{array}$$

The “cycles” is the number of cycles required to do all processing and the “frequency” is the frequency on which DFE is running. The time to stream the data to/from the DFE can be calculated using the next formula:

(2) $$\begin{array}{c} T_{\text{PCIe}}=\max\left(\;\frac{\text{BytesIn}}{\text{BandwidthIn}},\frac{\text{BytesOut}}{\text{BandwidthOut}}\;\right). \end{array}$$

From the point where acceleration gets bound with PCI Express bandwidth, the only thing that can be done is to try to compress input/output data. If this can be achieved, then it would make sense to add more pipes to the design.

## 6. Conclusion

In this paper, the implementation of the region-of-interest based image segmentation algorithm for breast mammograms on the DFE is proposed. The experimental results showed that there was a significant speedup in algorithm execution on DFE compared to the general-purpose processor. The experiments were performed on over two types of breast mammogram images with different resolutions. The results showed that, with better image resolution (i.e., with more data per image to process), the acceleration is greater. Also, there were several configurations of the DFE which were implemented for testing purposes and discussed in detail. The experimental results showed that the acceleration of algorithm execution goes near seven times for some DFE configurations.

Further work on this research may be in implementing other stages of the procedure for breast cancer detection from mammogram images on the DFE and exploring those acceleration results. It would be interesting to try to accelerate algorithms for identification of suspicious mass regions of breast mammograms which take as input the results of the algorithm described in this paper.

## Figures and Tables

**Algorithm 1:**
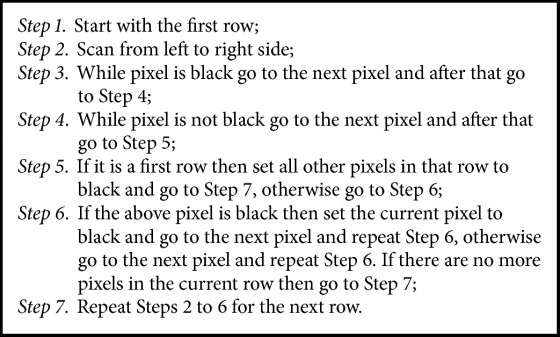
Background partition removal algorithm.

**Algorithm 2:**
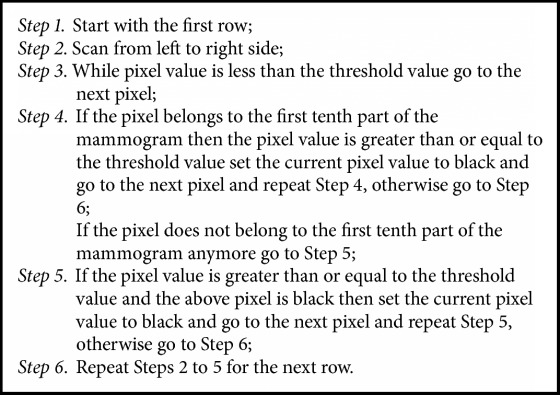
Pectoral muscle removal algorithm.

**Table 1:** FPGA resource usage per operator.

Operator	LUTs	FFs	BRAMs	DSPs
Counters	114	99	0	0
Comparison nodes	17	1	0	0
Deviation nodes	1225	1187	0	0
AND	1	1	0	0

**Table 2:** DFE resource usage.

	Total available resources	Total resources used	Used by kernels	Used by manager	Stray resources
LUTs	207360	44784 (21.60%)	26006 (12.54%)	18081 (8.72%)	11 (0.01%)
FFs	207360	52447 (25.29%)	26660 (12.86%)	24664 (11.89%)	94 (0.05%)
BRAMs	324	84 (25.93%)	11 (3.4%)	71 (21.91%)	0 (0%)
DSPs	192	21 (10.94%)	21 (10.94%)	0 (0%)	0 (0%)
